# Salidroside protects against myocardial infarction via activating MIF-mediated mitochondrial quality control

**DOI:** 10.1186/s13020-025-01076-3

**Published:** 2025-02-28

**Authors:** Baiyang You, Jie Zhang, Chuyan Yang, Yaoshan Dun, Dake Qi, Yuqiong Long, Jing Cheng, Yuan Lin, Nanjiang Zhou, Tanghao Zeng, Jie Dong, Suixin Liu

**Affiliations:** 1 Division of Cardiac Rehabilitation, Department of Physical Medicine and Rehabilitation, Xiangya Hospital Central South University, Changsha, Hunan China; 2 National Clinical Research Center for Geriatric Disorders, Xiangya Hospital Central South University, Changsha, Hunan China; 3 Department of Physical Medicine and Rehabilitation, Xiangya Hospital Central South University, Jiangxi (National Regional Center for Neurological Diseases), China; 4 Department of Rehabilitation Medicine, Jiangxi Provincial People’s Hospital, Clinical College of Nanchang Medical College, First Affiliated Hospital of Nanchang Medical College, Jiangxi, China; 5 College of Pharmacy, University of Manitoba, Winnipeg, MB Canada; 6 Department of Cardiovascular, Shenzhen District Yantian People’s Hospital, Shenzhen, Guangdong China

**Keywords:** Salidroside, Macrophage migration inhibitory factor, Myocardial infarction, Cardiomyocyte apoptosis, Mitochondrial quality control

## Abstract

**Background:**

Salidroside is a potential therapeutic agent for myocardial infarction (MI), exerting therapeutic effects on macrophage migration inhibitory factor (MIF)-regulated mitochondrial quality control. Our aim was to explore the mechanism through which the MIF pathway regulates salidroside-mediated resistance to hypoxia-induced cardiomyocyte apoptosis.

**Methods:**

Ligation surgery of the left anterior descending branch of the coronary artery was employed to establish a myocardial infarction mouse model. Salidroside at low and high doses was administered to the mice for 4 weeks after the surgery. Cardiac function was evaluated via echocardiography. Morphological changes, apoptosis, and mitochondrial damage in the myocardium were examined. For the cell experiments, cardiomyocytes were treated with salidroside under oxygen‒glucose deprivation (OGD) conditions and were either treated with recombinant MIF (rMIF) or transfected with *Mif*-siRNA. Subsequently, mitochondrial quality control and apoptosis were assessed.

**Results:**

Salidroside enhanced mitochondrial quality control in MI model mice, mitigated apoptosis and improved cardiac dysfunction. Transmission electron microscopy indicated that there were fewer damaged mitochondria in the salidroside-treated mice compared with the control mice. MIF and downstream mitochondrial quality control pathways were activated in the mice treated with salidroside. Consistently, the cell experiments demonstrated that salidroside and rMIF alleviated apoptosis, improved impaired mitochondrial quality control in OGD-induced cells and activated MIF signaling in OGD-induced cells. However, these effects of salidroside were partially blocked by *Mif*-siRNA transfection.

**Conclusion:**

Salidroside alleviated myocardial apoptosis and ameliorated cardiac dysfunction in MI model mice through the MIF pathway and downstream mitochondrial quality control.

**Supplementary Information:**

The online version contains supplementary material available at 10.1186/s13020-025-01076-3.

## Introduction

Myocardial infarction (MI) is characterized by the death of myocardial cells due to sustained ischemia [1]. Despite the widespread implementation of evidence-based therapeutic approaches, including percutaneous coronary intervention, coronary artery bypass grafting, MI remains a major public health concern. The high mortality and morbidity rates of MI impose significant economic burden, and patients may encounter complications, such as ischemia‒reperfusion injury and coronary restenosis after treatment [2, 3]. Therefore, it is necessary to explore additional therapeutic strategies to inhibit cardiomyocyte apoptosis in MI.

Following MI, the non-infarcted myocardial tissue undergoes adaptation to compensate for the loss of functional myocardium [4]. Pathological hypertrophy can cause individual cardiomyocytes to elongate, leading to ventricular chamber dilation, which impairs contractile function. The outcome of pathological cardiac hypertrophy includes a reduction in cardiac output, an increased risk of heart failure, and eventual progression to heart failure. Reversing this maladaptive process has become a key therapeutic goal. Targeting the renin–angiotensin–aldosterone system involves using mineralocorticoid receptor antagonists to reduce the left ventricular mass index, restore ventricular compliance, and decrease overall mortality [5]. However, research has shown that pathological cardiac hypertrophy is primarily regulated by transcription factors, non-coding transcripts, and even histone modifications [6–8]. Small molecule drugs effectively modulate these gene expression networks [9]. This study aims to identify potential intervention targets and drugs for mitigating post-infarction myocardial hypertrophy.

Salidroside (SAL) is the principal active component extracted from various *Rhodiola* roots, is well-known for its adaptogenic properties and has been used traditionally to alleviate altitude sickness [10]. Owing to its proangiogenic, prosurvival, anti-inflammatory, and antioxidant properties, salidroside is considered a promising treatment for ischemic heart disease [11]. Studies have demonstrated that salidroside can reduce hypoxia-induced cardiomyocyte apoptosis [12–14] by activating the HIF-1α/VEGF pathway [15] and restoring mitochondrial function [12]. Our previous research indicated that, similar to regular exercise, *Rhodiola* has cardioprotective effects on exhaustive exercise-induced myocardial injury [16–18]. Nevertheless, the effects of salidroside on MI need to be verified, and the underlying mechanisms warrant further investigation.

Macrophage migration inhibitory factor (MIF) is a multifunctional cytokine associated with several inflammatory and immune diseases [19]. MIF has been identified as a critical target for the neuroprotective effects of exercise in ischemic stroke [20]. Considering that salidroside enhances the benefits of exercise, and exerts exercise-like effects, our previous study demonstrated that *Rhodiola* acts on the MIF pathway and downstream lipid metabolism in hepatocytes [21]. Therefore, we hypothesize that salidroside protects cardiomyocytes against MI by activating the MIF pathway.

MIF binds to cluster of differentiation 74 (CD74) or alternative receptors [22] and is capable of enhancing angiogenesis, attenuating apoptosis, promoting cardiomyocyte survival, inhibiting fibrosis, and preserving heart function in the ischemic myocardium. This effect is mediated via the miR-133a-3p/AKT pathway [19, 23]. MIF also activates adenosine monophosphate-activated protein kinase (AMPK) [24], which mitigates acute myocardial injury through mitochondrial quality control (MQC), encompassing mitochondrial biogenesis, fission, fusion, and mitophagy [25–28]. A recent study demonstrated that when the myocardium is damaged as a result of exposure to side-stream smoke, activation of the MIF pathway maintains a stable mitochondrial content, enhances mitochondrial autophagy, improves myocardial systolic function, and alleviates cardiomyocyte apoptosis [29]. Based on these findings we hypothesize that salidroside improves the prognosis of MI through the MIF pathway-mediated downstream mitochondrial homeostasis. The subsequent study will verify this deduction.

## Materials and methods

### Animals

Eight-week-old male C57BL/6 J mice (20 ± 2 g) were acquired from the Laboratory Animal Centre of Xiangya Medical School (Changsha, CN). The mice were housed under controlled conditions (22 ± 2 °C temperature, 45–55% humidity, 12-h light‒dark cycle), with refreshed padding every three days, and ad libitum access to food and water.

All animal experiments conformed to the Guide for the Care and Use of Laboratory Animals and were approved by the Medicine Animal Welfare Committee of Xiangya Medical School, Central South University (Changsha, CN) (approval ID: CSU-2022-0281).

### Groups

Mice that underwent MI surgery were treated with salidroside to observe its therapeutic effects on cardiomyocytes. The mice were divided into two groups: the sham group (with only chest opening and no ligation of the left anterior descending artery) and the myocardial infarction (MI) group. After adaptive feeding for 1 week, these two groups were further randomly divided into four subgroups (n = 8): the control (Ctrl), enalapril maleate (Enalapril, 20 mg/kg per day), low-concentration salidroside (Sal-L, 100 mg/kg per day), and high-concentration salidroside (Sal-H, 200 mg/kg per day) groups.

The Salidroside intake of a mice in the Sal-L group and Sal-H was 100 mg/kg and 200 mg/kg, respectively according to Xue et al.’s study [30] and our previous study [31]. Salidroside powder (R093379, Rhawn, Shanghai, CN), whose purity is 98%, was diluted to the desired concentrations with distilled water. Considering mice consumed ~ 7.2 mL of water/day, Sal-L and Sal-H concentrations were adjusted to 30 mg/100 mL and 60 mg/100 mL, respectively. Water consumption did not differ among the all groups. Enalapril (55156ES80, Yeasen, Shanghai, CN), a first-line treatment for heart failure, served as a positive control and was administered orally at 20 mg/kg/day.

For the assessment of short-term apoptosis, salidroside was administered from day 0 to day 7 after MI. On day 7 after the surgery, the mice were sacrificed, and heart samples were collected for TUNEL staining and western blotting.

For the evaluation of long-term efficacy, salidroside was administered from day 0 to day 28 after MI. On Day 0 and Day 28, echocardiography was conducted in the sham and MI groups to assess cardiac function at baseline and post-intervention. On day 28 post-intervention, the mice were sacrificed and weighed. Subsequently, heart samples were excised, weighed, and utilized for further experiments.

### Myocardial infarction model

The myocardial infarction (MI) model was established by ligating the left anterior descending (LAD) coronary artery, following the method described in study of Zhijun Lin et. al. with appropriate modifications [32]. Mice were anesthetized through inhalation of 2% isoflurane and then underwent endotracheal intubation and mechanical ventilation under continuous anesthesia (1.5% isoflurane via endotracheal intubation). The ventilation parameters were set at a tidal volume of 1.8 mL, an inhalation-to-respiration ratio of 5:4, and 120 breaths per minute. Subsequently, the thorax was shaved and disinfected. After left thoracotomy, the heart was partially exposed, and the left anterior descending artery was ligated 2 mm below the left auricle using a 10–0 silk suture. Finally, the incision was sutured, and 0.1 mg/kg buprenorphine was administered. Intraoperative observations of the color changes in the anterior wall below the ligation point and echocardiographic results indicated that the mice had experienced a comparable degree of MI.

### Echocardiography

Serial echocardiography (Mindray Inc., Nanjing, CN) was employed to evaluate left ventricular function. The mice underwent chest depilation and were then anesthetized with 2% isoflurane, placed in the left lateral decubitus position, and after the coupling agent was applied to the chest area, the probe was placed thereon for imaging. M-mode recordings of the left ventricle were obtained at the level of the papillary muscle in the parasternal short-axis view. Once the left ventricular internal diameter at end-diastole (LVIDd) and the left ventricular internal diameter at end-systolic (LVIDs) were measured, the ejection fraction (EF) and fractional shortening (FS) were calculated automatically.

### Myocardial histopathology

The excised heart samples were embedded in paraffin and then sliced along the short axis from the atrium to the apex. The parts beneath the ligated knots in the MI groups and the corresponding parts in the sham groups were retained for Masson’s trichrome staining.

Masson’s trichrome staining was carried out in accordance with the manufacturer’s protocol (G1006, Servicebio, Wuhan, CN). After Masson’s trichrome staining, the collagen volume fraction was calculated using ImageJ (NIH, MD, US) to assess the degree of fibrosis in both the sham and MI groups.

TTC (2,3,5-Triphenyltetrazolium chloride) staining was performed according to the manufacturer’s protocol (GP1047, Servicebio, Wuhan, CN). Stained slices were photographed and the images analyzed to determine the infarct area. Normal myocardium appears red, while infarcted tissue remains unstained.

Wheat germ agglutinin staining was carried out in accordance with the manufacturer’s instructions (1:200, G1730, Servicebio) to determine the cross-sectional area of the myocardium in the MI groups, and the analysis was conducted via ImageJ (NIH, MD, US) software.

### Molecular docking

The salidroside ligand structure was obtained from the PubChem database, and the protein structure was selected from the Protein Data Bank (PDB) with a high-resolution crystal structure (1.17 Å) of the MIF complex (PDB ID: 6B1K). Chimera was used to add hydrogen atoms and calculate charges for both the protein and the ligand [33]. Molecular docking was performed via DOCK6.9 [34]. The binding site was defined on the basis of the location of inhibitor 3a in the crystal structure, with a docking region encompassing a 5 Å radius around inhibitor 3a. Given that inhibitor 3a forms hydrogen bonds with multiple chains of the trimeric protein in the crystal structure, the trimeric MIF protein, rather than the monomeric form, was used for both molecular docking and molecular dynamics (MD) simulations. The pose with the highest docking score from DOCK6 was selected as the initial pose for subsequent analyses. Protein‒ligand interactions were analyzed in 2D via LigPlot + v2.2 [35] and in 3D via the PLIP web tool [36].

### Molecular dynamics simulations

The docked complex was prepared via AmberTools23 [37]. The AM1-BCC charges for salidroside were computed via the Antechamber module, and the receptor protein was parameterized via the Amber ff19SB force field [38], with water molecules modeled via the TIP3P force field [39]. Subsequent energy minimization, 100 ps heating simulation, 100 ps pressure equilibration simulation, and 200 ns production run were conducted via Amber22. Structural analyses of the MD trajectories, including RMSD, RMSF, and hydrogen bond analysis, were carried out via cpptraj from AmberTools. The MM-GBSA binding free energy calculations and per-residue energy decomposition were performed via MMPBSA.py.

### Surface plasmon resonance (SPR) assays

SPR assays (Biacore T200, Cytiva) were conducted to examine the binding affinity of salidroside to MIF. The CM5 sensor chip was activated, and subsequently, the MIF protein was injected onto the active channel surfaces to immobilize the ligand. Salidroside was diluted with the running buffer and injected into the active and reference channels at a flow rate of 50 μL/min. The dissociation time was 60 s. Appropriate and consecutive concentrations (at least 5 concentrations) were selected to carry out kinetic 1:1 binding or steady-state fitting.

### Immunofluorescence analysis

The sections were incubated at 4 °C overnight with a primary antibody against MIF (1:100, Proteintech, USA) t, and subsequently stained with a donkey anti-rabbit secondary antibody (Abcam, UK) at room temperature for 1 h. Finally, the sections were sealed with mounting medium containing 4',6-diamidino-2-phenylindole (DAPI, G1012, Servicebio) and imaged using a fluorescence microscope (DM3000 LED, Leica, Germany).

### TUNEL staining

A terminal deoxynucleotidyl transferase dUTP nick end labeling (TUNEL) assay was employed to assess the degree of myocardial apoptosis. TUNEL staining was carried out in accordance with the instructions of the TUNEL fluorescence FITC kit (G1501, Servicebio). The nuclei were then counterstained with DAPI. The peripheral infarct areas were selected for assessment. Images of each section were captured using a fluorescence microscope.

### Transmission electron microscopy

A transmission electron microscope (Tecnai G2 Spirit, FEI, USA) was employed to detect the morphological alterations of mitochondria in heart samples. The tissues (approximately 1 mm3 in size) were successively fixed in 2.5% glutaraldehyde and 1% osmium, dehydrated in graded ethanol, embedded in epoxy resin, sliced to a thickness of 50–100 nm, and subsequently stained with uranyl acetate and lead citrate. Then, the sections were observed under a transmission electron microscope, and the degree of mitochondrial damage was quantified by calculating the area of damaged mitochondria in relation to the total area of the field in the myocardium.

### Analysis of mitochondrial mass

Total DNA was extracted from heart samples by using a DNeasy Kit (Qiagen). To determine the mitochondrial content, quantitative real-time PCR was employed to measure the amounts of mitochondrial DNA (mtDNA, MtCO3 oligos) and nuclear DNA (nucDNA, SDHA, succinate dehydrogenase complex subunit A). Subsequently, the ratio of mtDNA to nuclear DNA was calculated. The specific primers utilized are presented in Table 1.

**Table 1:** Primer sequences

Gene	Primer
Ms-DNA-MtCO3	F-GCAGGATTCTTCTGAGCGTTCT R-GTCAGCAGCCTCCTAGATCATGT
Ms-DNA-Sdha	F-TACTACAGCCCCAAGTCT R-TGGACCCATCTTCTATGC

### Cell experiments

Cardiomyocytes (H9C2 cells, Zishan Biotechnology Co., Ltd., Wuhan, CN) were cultured in complete medium consisting of 10% fetal bovine serum and 1% penicillin/streptomycin (Gibco, New York, USA) in Dulbecco’s modified Eagle’s medium (DMEM). The interventions are described as follows.

1. Logarithmically growing cells were seeded in 6-well plates at 1 × 10^5^ cells per well. After that, the cells were subjected to oxygen‒glucose deprivation (OGD) or a normal control (Ctrl) and treated with phosphate-buffered saline (PBS) or different concentrations of salidroside (10, 20, or 50 μg/mL) for 24 h. For OGD treatment, the cells were cultured in glucose-free DMEM in a tri-gas chamber (YCP-150, Huaxi, Changsha, CN) under hypoxic conditions (1% O_2_, 5% CO_2_, 94% N_2_). In the Ctrl group, the cells were cultured with high-glucose DMEM in a normoxic chamber (95% air and 5% CO_2_). Then, the cells were prepared for cell counting kit-8 (CCK-8) assays, western blotting, or flow cytometry.
2. After being seeded in 6-well plates, the cells were subjected to OGD or Ctrl conditions, si*Mif* or siNC transfection, and treatment with PBS or 200 ng/mL recombinant MIF (rMIF; PeproTech, NJ, USA) for 24 h. Then, the cells were prepared for western blotting, mitochondrial labeling, flow cytometry, and ATP content measurement.
3. After being seeded in 6-well plates, the cells were subjected to OGD or Ctrl conditions, PBS or 50 μg/mL salidroside treatment, si*Mif* or siNC transfection, and PBS or rMIF treatment for 24 h. Then, the cells were prepared for western blotting, mitochondrial labeling, flow cytometry, and ATP content measurement.

### siRNA transfection

After being seeded in 6-well plates, cardiomyocytes were transfected with *Mif* siRNA or negative control siRNA (RiboBio, Guangzhou, CN) in the presence of Lipo3000 (Invitrogen, CA, USA). The sequence of the *Mif* siRNA used was CTTTTAGTGGCACGAGCGA.

### CCK-8 assay

A CCK-8 assay was performed to detect cell viability at 4, 12, and 24 h. Cardiomyocytes were seeded at a density of 5 × 10^3^ cells/well in 96-well plates and then subjected to different treatments. After the CCK-8 solution was added to each well, the cells were cultured at 37 °C, and then, the absorbance at 450 nm was measured with a microplate reader.

### Western blotting

Proteins were extracted from heart samples or cardiomyocytes with radioimmunoprecipitation assay (RIPA) buffer (Beyotime, Nanjing, CN) and phenylmethanesulfonyl fluoride (PMSF; Beyotime). A bicinchoninic acid (BCA) protein assay kit (Beyotime) was subsequently used to measure the protein concentration. After sodium dodecyl sulfate‒polyacrylamide gel electrophoresis (SDS‒PAGE), electrophoretic transfer, and nonspecific binding blocking, the transferred proteins were incubated overnight with primary antibodies against GAPDH (1:5000, 10494-1-AP, Proteintech), Bcl-2 (1:2000, ab182858, Abcam), Bax (1:2000, ab32503, Abcam), cleaved caspase-3 (1:1000, #9661, CST), MIF (1:1000, ab187064, Abcam), AMPK (1:1500, 66536-1-Ig, Proteintech), p-AMPK (1:1000, ab23875, Abcam), microtubule-associated protein 1 light chain 3 II (LC3II, 1:1000, 14600-1-AP, Proteintech), PTEN-induced putative kinase 1 (PINK1, 1:1000, 23274-1-AP, Proteintech), BCL2-interacting protein 3 (BNIP3, 1:1000, ab109362, Abcam), Optic Atrophy 1 (OPA1, 1:2000, 27733-1-AP, Proteintech), and Mitofusin 1 (MFN1, 1:1000, 2398-1-AP, Proteintech), followed by incubation with the corresponding Band intensity was analyzed using a gel documentation system (Bio-Rad, Hercules, CA, USA) and normalized to that of GAPDH.

### Flow cytometry

The percentage of apoptotic cardiomyocytes was determined by flow cytometry in accordance with the instructions of the Annexin V-FITC/PI Cell Apoptosis Detection Kit (G1511, Servicebio). Briefly, cardiomyocytes were digested with EDTA-free trypsin, washed twice with cold PBS, centrifuged each time, resuspended in cold 1 × binding buffer, adjusted to a concentration of 1–5 × 10^6^/mL, dual stained with Annexin V-FITC and PI in the dark for 8–10 min, resuspended in cold 1 × binding buffer, and detected using a flow cytometer (NL3000, Cytek, CA, USA).

### Mitochondrial labeling

After treatment, the cells were incubated with preheated MitoTracker Red FM staining working solution (1:10,000, 40741ES50, Yeasen, Shanghai, CN) at 37 °C for 30 min and then examined under a fluorescence microscope.

### ATP content

The ATP content of cardiomyocytes was determined by the phosphomolybdic acid colorimetric method in accordance with the manufacturer's protocol (A095-1-1, Nanjing Jiancheng Bioengineering Institute, Nanjing, CN).

### Statistical analysis

The data are presented as the means ± SDs. Statistical analysis was carried out through one-way ANOVA and the Bonferroni multiple comparisons test using Prism 6 (GraphPad, Inc., CA, USA). A two-sided P value < 0.05 was regarded as statistically significant.

## Results

### Salidroside alleviates cardiac dysfunction and myocardial apoptosis in myocardial infarction model mice

**Fig. 1:**
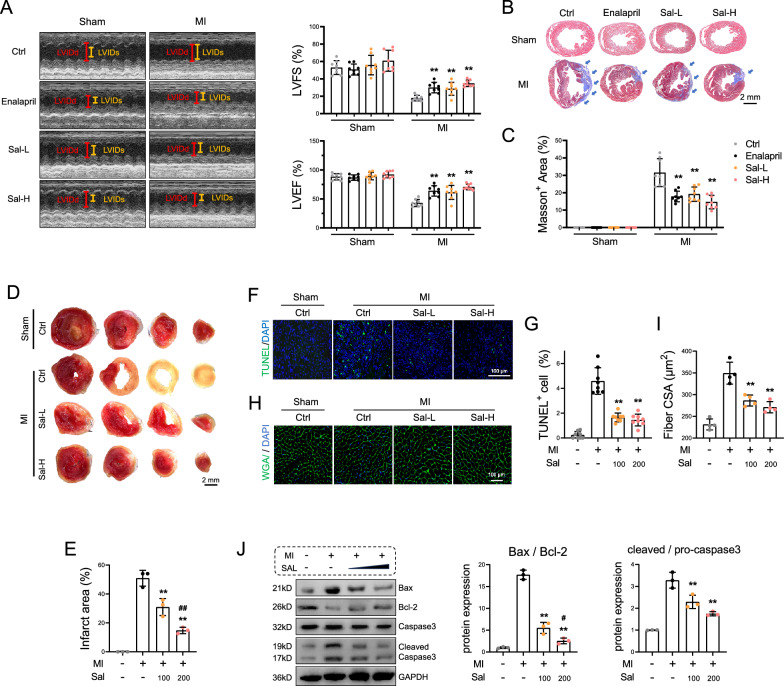
Salidroside alleviates cardiac dysfunction and myocardial apoptosis in myocardial infarction mice. **A** Mice with or without undergoing MI surgery were administered with vehicle control (Ctrl), enalapril (20 mg/kg), or salidroside at a low dose (SAL-L, 100 mg/kg) or a high dose (SAL-H, 200 mg/kg) once per day. On day 28 post surgery, M-mode echocardiograms, the ejection fraction, and the shortening fraction were examined via echocardiography (n = 8). **B** Masson’s trichrome staining of the myocardium postintervention were performed, the blue arrows indicate fibrosis area. **C** The proportion of the Masson-positive area was quantified; scale bar = 2 mm (n = 8). **D** Representative images of TTC staining were performed on day 7 post surgery. **E** The infarct area of TTC-stained heart was quantified, scale bar = 2 mm (n = 3).** F** Images of TUNEL staining were obtained on day 7 post surgery, scale bar = 100 μm. **G** The percentage of TUNEL^+^ cells was analyzed (n = 8). **H** Images of wheat germ agglutinin (WGA) were obtained on day 28 post surgery, scale bar = 100 μm. **I** Quantitative analysis of the cross-sectional area (n = 4). **J** Western blot analysis of the protein expression of Bcl-2, Bax and caspase 3 (n = 3). The data are presented as the means ± SDs. Statistical analysis was performed via one-way ANOVA followed by Bonferroni’s multiple comparisons test. *P < 0.05 vs. the MI-Ctrl group; **P < 0.01 vs. the MI-Ctrl group; ^#^*P* < 0.05 vs. the MI-Sal-L group

A low concentration of salidroside (Sal-L), or a high concentration of salidroside (Sal-H) was orally administered to the mice that underwent MI surgery for 4 weeks. Enalapril (ACEI class), was selected as positive controls. HE staining revealed that salidroside did not have a toxic effect on the liver, kidney or other organs of the mice (Fig. S1). The echocardiography results showed that salidroside increased the ejection fraction and fraction shortening in the Sal-L and Sal-H groups compared with those in the Ctrl group that underwent MI surgery (Fig. 1A). Masson’s trichrome staining revealed that salidroside did not change the heart structure of the mice in the sham group. Salidroside reduced the area of fibrosis in the MI groups, as evidenced by a smaller Masson-positive area (Fig. 1B–C). Additionally, we observed a reduction in the infarct area and the number of TUNEL-positive cells in both Sal groups on day 7 post surgery (Fig. 1D–G). Finally, at day 28 post surgery, salidroside administration largely decreased the cross-sectional area of myocardial fibers in myocardial infarction model mice (Fig. 1H–I), which was accompanied by the following changes in apoptosis markers: elevated levels of Bcl-2 and reduced Bax and cleaved caspase 3 levels (Fig. 1J). These results indicate that both doses of salidroside improve myocardial apoptosis, structure, and function after MI. Furthermore, Sal-H administration had better therapeutic effects than Sal-L administration in some aspects.

### Salidroside activates MIF signaling and decreases mitochondrial damage in myocardial infarction model mice

**Fig. 2:**
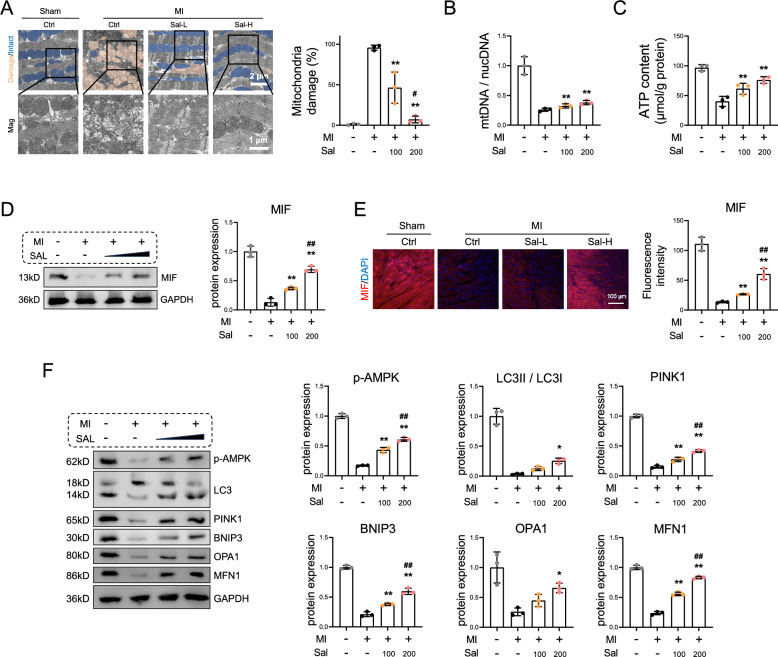
Salidroside activated MIF signaling and decreased mitochondrial damage in myocardial infarction model mice. **A** Salidroside was administered at various concentrations following MI surgery, and heart tissue samples were collected. The mitochondrial ultrastructure was observed via transmission electron microscopy, and damaged mitochondria were quantified. The yellow or blue regions indicate damaged mitochondria or intact mitochondria, respectively, scale bar = 2 or 1 μm. **B** The ratio of mtDNA to nuclear DNA was determined via quantitative real-time PCR.** C** ATP content was measured.** D** Western blot analysis of the expression of MIF. **E** Representative images of MIF immunofluorescence staining and data analysis of heart sections. The cell nuclei were stained blue with DAPI, and MIF was stained red, scale bar = 100 μm. **F** Western blot analysis of the expression of p-AMPK, LC3I, LC3II, PINK1, BNIP3, OPA1, and MFN1 were performed. Statistical analysis was performed via one-way ANOVA followed by Bonferroni’s multiple comparisons test. n = 3. *P < 0.05 vs. the MI-Ctrl group; **P < 0.01 vs. the MI-Ctrl group; ^#^*P* < 0.05 vs. the MI-Sal-L group; ^##^*P* < 0.01 vs. the MI-Sal-L group

To explore the mechanism underlying the cardioprotective effects of salidroside, we investigated mitochondrial homeostasis and MIF-mediated MQC. As depicted in Fig. 2A, MI led to extensive mitochondrial swelling and loss of cristae, while treatment with salidroside mitigated mitochondrial damage. Additionally, the Sal groups exhibited higher mtDNA expression and ATP content compared to the Ctrl group (Fig. 2B–C). Furthermore, salidroside upregulated MIF in contrast to the Ctrl group, and high-concentration salidroside promoted the expression of MIF more significantly than low-concentration salidroside did (Fig. 2D–E). Next, we detected the expression of p-AMPK, mitophagy markers (LC3II/LC3I, PINK1, and BNIP3) and proteins related to mitochondrial quality control (OPA1 and MFN1). The results indicated that high concentrations of salidroside evidently promoted AMPK phosphorylation and increased the levels of these proteins (Fig. 2F).

### MIF could be the target by which salidroside protects the myocardium

**Fig. 3:**
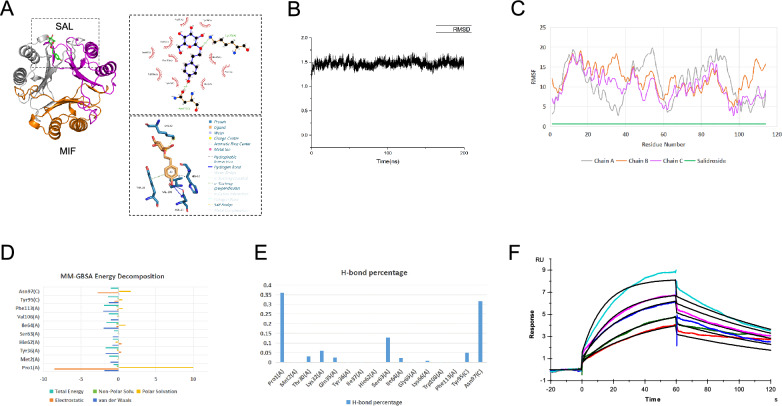
MIF could be the target by which salidroside protects the myocardium. **A** Docking and interaction analysis of salidroside with the MIF protein. **B** RMSD plot of the complex, indicating its stability over time. **C** RMSF plot showing the flexibility of residues in the MIF protein and salidroside. **D** Top ten residue contributions to the binding free energy (MM-GBSA) of the complex, computed via energy decomposition analysis. **E** Hydrogen bond occupancy analysis between salidroside and MIF residues over the simulation trajectory, with MIF chain identifiers in parentheses. The energy unit is kcal/mol, and the RMSD/RMSF units are in angstroms (Å). **F** SPR assay for MIF protein and salidroside

The docking results from DOCK6 indicated that the primary interactions between salidroside and MIF involve hydrogen bonds formed with Lys32 on chain A and with Asn97 on chain C (Fig. 3A). Additionally, the 3D interaction analysis revealed further intricate details. Specifically, the hydroxyl groups of salidroside form hydrogen bonds with Asn97 on chain C, facilitating strong binding between the ligand and the receptor. The aromatic ring of salidroside establishes notable hydrophobic interactions with His62 and Val106 on chain A. Moreover, the π‒electron cloud of the aromatic ring of salidroside engages in vertical π‒π stacking interactions with the side chain of Tyr95 on chain C. Notably, 3D interaction analysis demonstrated that the Lys32 residue on chain A primarily forms salt bridges with the sugar ring of salidroside rather than conventional hydrogen bonds. These interactions collectively contribute to the increased binding stability between the ligand and the receptor. The docking score for the salidroside-MIF complex was − 45.254684 kcal/mol, indicating strong binding affinity.

The binding mechanism of the complex was further validated through molecular dynamics simulations. The RMSD results demonstrated that the complex trajectory converged to stability, indicating that salidroside remained stably bound in the MIF binding pocket over the 200 ns simulation period (Fig. 3B). The RMSF analysis revealed that the trimeric MIF protein experienced no significant structural fluctuations over the 200 ns simulation, and the presence of salidroside contributed to the stabilization of specific regions of the protein, such as around Lys32 on chain A and Tyr95 on chain C. The RMSF of salidroside also remained consistently low, suggesting stable binding (Fig. 3C). The MM-GBSA binding free energy for the salidroside-MIF complex was calculated to be -28.7231 kcal/mol. Residue energy decomposition analysis revealed a shift in dominant interactions compared with the initial docking pose; salidroside did not bind primarily to Lys32. Instead, within the trimeric MIF, salidroside exhibited strong interactions with residues such as Pro1, Met2, Tyr36, His62, Ser63, Ile64, Val106, and Phe113 on chain A and Tyr95 and Asn97 on chain C (Fig. 3D). Hydrogen bond occupancy analysis revealed that Pro1 on chain A and Asn97 on chain C formed the most hydrogen bonds with the ligand, each exceeding 30% occupancy. Other significant hydrogen bonds involved Ser63 and Lys32 on chain A and Tyr95 on chain C (Fig. 3E). Interestingly, Lys32 formed hydrogen bonds only 6% of the time, possibly because of its primary interaction with the sugar ring of salidroside, where numerous hydroxyl groups may participate in fluctuating interactions with the aqueous solvent, leading to instability. These interaction analyses align with previously reported key residues in the MIF binding pocket [40], supporting the potential of salidroside as an MIF inhibitor. To further determine the kinetics between salidroside and the MIF protein, a surface plasmon resonance (SPR) assay was performed. Under the conditions of this experiment, the affinity constant (K_D_) between the MIF protein and salidroside was 1.188 × 10^–5^ (Fig. 3F).

### Salidroside upregulates MIF signaling and attenuates oxygen‒glucose deprivation (OGD)-induced cardiomyocyte apoptosis

**Fig. 4:**
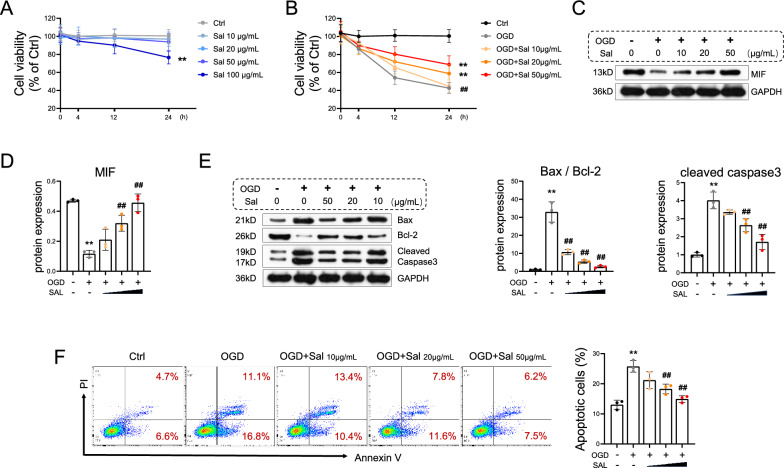
Salidroside upregulates MIF signaling and attenuates oxygen‒glucose deprivation (OGD)-induced cardiomyocyte apoptosis. **A** Cell viability was measured via a CCK-8 assay after cardiomyocytes were treated with different concentrations (0, 10, 20, 50, or 100 μg/mL) of salidroside. **B** After treatment with salidroside or PBS with or without oxygen‒glucose deprivation (OGD) for 24 h, the viability of cardiomyocytes was determined via CCK-8 assays. **C** Western blot of the expression of MIF was performed. **D** MIF protein expression is analyzed. **E** Western blot and analysis of the expression levels of Bax, Bcl-2, and caspase 3 were performed. **F** Cardiomyocyte apoptosis was detected and quantified by flow cytometry. The data are presented as the means ± SDs. Statistical analysis was performed via one-way ANOVA followed by Bonferroni’s multiple comparisons test. n = 3. *P < 0.05 vs. the Ctrl group, **P < 0.01 vs. the Ctrl group, ^#^*P* < 0.05 vs. the OGD group, ^##^*P* < 0.01 vs. the OGD group

The toxicity of salidroside to cardiomyocytes at various doses (0, 10, 20, 50, and 100 μg/mL) was evaluated through a CCK-8 assay. Compared with the control, salidroside at 10, 20, and 50 μg/mL had no impact on cardiomyocyte survival when incubated for 4, 12, and 24 h, while 100 μg/mL of salidroside led to a reduction in cell viability (Fig. 4A). To explore the function of salidroside in vitro, cardiomyocytes were treated with different concentrations of salidroside or PBS, followed by oxygen‒glucose deprivation (OGD) or control conditions for 24 h. Notably, cell viability was lower in the OGD group than in the Ctrl group but increased with the rising concentrations of salidroside (Fig. 4B). In contrast to the Ctrl group, OGD downregulated MIF. However, MIF was enhanced in a dose-dependent manner by the supplementation of salidroside (Fig. 4C–D). Additionally, the administration of salidroside inhibited the OGD-induced increase in Bax/Bcl-2 and cleaved caspase 3 (Fig. 4E). Moreover, the same trend was observed in the percentage of apoptotic cells, as indicated by annexin V/PI staining and flow cytometry (Fig. 4F).

### Effects of MIF on oxygen‒glucose deprivation (OGD)-induced apoptosis and mitochondrial dysfunction in cardiomyocytes

**Fig. 5:**
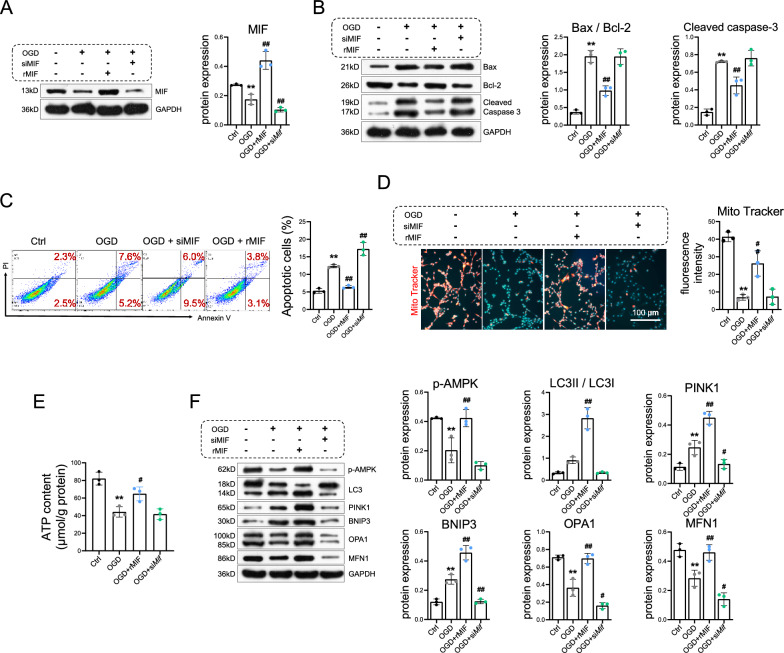
Effects of MIF on oxygen‒glucose deprivation (OGD)-induced apoptosis and mitochondrial dysfunction in cardiomyocytes. **A** Cardiomyocytes were subjected to oxygen‒glucose deprivation (OGD) or control conditions and transfected with si*Mif* or incubated with recombinant MIF (rMIF) for 24 h. Then, western blot analysis of the expression of MIF was performed. **B** Western blot analysis of the expression of Bax, Bcl-2, and caspase-3 was performed. **C** Cardiomyocyte apoptosis was detected and quantified by flow cytometry. **D** Mitochondria were quantified by mitochondrial labeling, and the data were analyzed. Scale bar = 100 μm. **E** ATP content was measured. **F** Western blot analysis of p-AMPK, LC3-I, LC3-II, PINK1, BNIP3, OPA1, and MFN1 was performed. The data are presented as the means ± SDs. Statistical analysis was performed via one-way ANOVA followed by Bonferroni’s multiple comparisons test. n = 3. *P < 0.05 vs. the Ctrl group, **P < 0.01 vs. the Ctrl group, ^#^*P* < 0.05 vs. the OGD group, ^##^*P* < 0.01 vs. the OGD group

Cardiomyocytes were subjected to si*Mif* transfection or recombinant MIF treatment followed by OGD for 24 h. Consistent with the results presented in Fig. 4, OGD suppressed the expression of MIF, induced apoptosis (i.e., upregulated Bax and cleaved caspase-3, downregulated Bcl-2, and increased the percentage of apoptotic cells) in cardiomyocytes (Fig. 5A–C) and further disrupted mitochondrial homeostasis (Fig. 5D–F).

After cardiomyocytes were treated with rMIF under OGD conditions, the MIF protein was activated (Fig. 5A), and myocardial apoptosis was alleviated (Fig. 5B–C). Furthermore, rMIF enhanced the content of mitochondria and ATP levels (Fig. 5D–E). Additionally, rMIF treatment elevated mitochondrial quality control, as indicated by the upregulation of mitophagy markers and mitochondrial fission and fusion proteins (Fig. 5F). When cardiomyocytes were transfected with si*Mif* (Fig. S2), contrary effects were observed on MIF expression, myocardial apoptosis, and the extent of mitochondrial quality control, although the levels of cleaved caspase 3, the LC3II/LC3I ratio and ATP levels were not significantly changed (Fig. 5).

### Salidroside alleviates mitochondrial dysfunction and apoptosis in oxygen‒glucose deprivation (OGD)-induced cardiomyocytes via MIF signaling

**Fig. 6:**
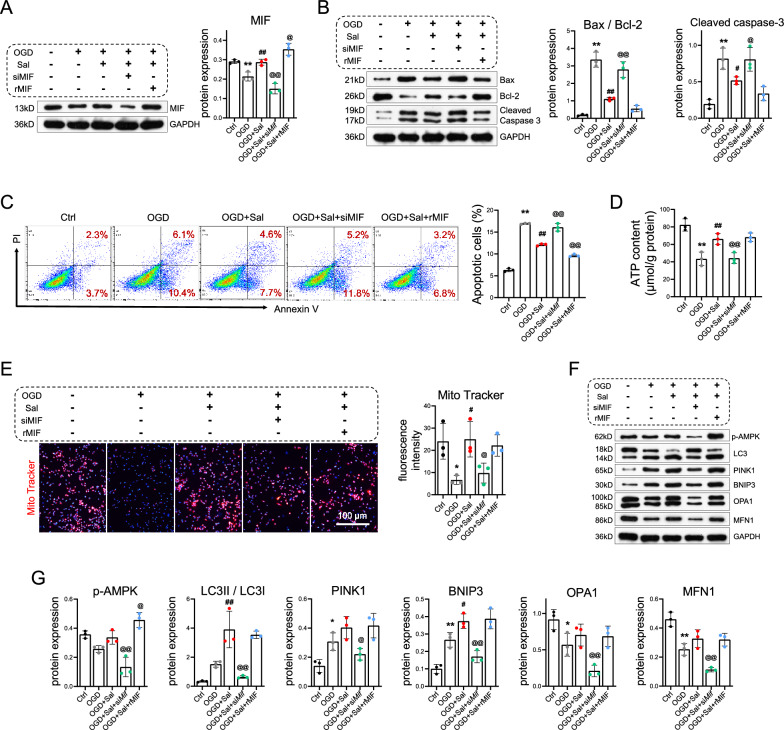
Salidroside alleviates mitochondrial dysfunction and apoptosis in oxygen‒glucose deprivation (OGD)-induced cardiomyocytes via MIF signaling. **A** Cardiomyocytes were transfected with si*Mif* or treated with recombinant MIF and then treated with salidroside or PBS with or without oxygen‒glucose deprivation (OGD) for 24 h. Proteins were subsequently harvested for western blotting to determine the level of MIF. **B** Western blotting was performed to determine the expression levels of Bax, Bcl-2, and cleaved caspase 3. **C** Cardiomyocyte apoptosis was detected and quantified by flow cytometry.** D** ATP content was measured. **E** Mitochondria were quantified by mitochondrial labeling. Scale bar = 100 μm.** F, G** Western blot analysis of the levels of p-AMPK, LC3I, LC3II, PINK1, BNIP3, OPA1, and MFN1 was performed. The data are presented as the means ± SDs. Statistical analysis was performed via one-way ANOVA followed by Bonferroni’s multiple comparisons test. n = 3. *P < 0.05 vs. the Ctrl group, **P < 0.01 vs. the Ctrl group, ^#^P < 0.05 vs. the OGD group, ^##^*P* < 0.01 vs. the OGD group, ^@^*P* < 0.05 vs. the Sal group, ^@@^*P* < 0.01 vs. the Sal group

To further confirm that MIF is a target of salidroside, cardiomyocytes were transfected with si*Mif* or treated with recombinant MIF, followed by treatment with salidroside (50 μg/mL) plus OGD for 24 h. Salidroside enhanced the expression of MIF; however, silencing MIF completely reversed this effect (Fig. 6A). Similarly, si*Mif* prevented the salidroside-induced decreases in Bax/Bcl-2, cleaved caspase 3 (Fig. 6B), as well as the proportion of apoptotic cells (Fig. 6C). Additionally, si*Mif* suppressed the salidroside-mediated increase in ATP levels (Fig. 6D) and mitochondrial content (Fig. 6E). It was evident that supplementation with rMIF had a synergistic effect with salidroside. Mechanistically, salidroside facilitated the activation of AMPK and downstream MQC, as indicated by the increases in p-AMPK, LC3-II/LC3-I, PINK1, BNIP3, OPA1, and MFN1, while si*Mif* treatment inhibited these increments (Fig. 6F).

## Discussion

The objective of this study was to explore the mechanism through which salidroside improves the prognosis of MI. The primary findings are as follows: (1) Salidroside activated the MIF pathway in the infarcted myocardium, decreased myocardial apoptosis, and enhanced cardiac function; (2) Under oxygen‒glucose deprivation conditions, salidroside activated the MIF pathway and downstream mitophagy and mitigating cardiomyocyte apoptosis, with recombinant MIF (rMIF) exerting similar effects; and (3) Silencing MIF abolished the protective effects of salidroside on cardiomyocytes. Collectively, these results suggest that the therapeutic effects of salidroside on MI are at least partially ascribed to the activation of MIF and downstream mitochondrial quality control.

The inflammatory response, oxidative stress, myocardial apoptosis, and myocardial fibrosis are well-documented hallmarks of MI pathophysiology, which lead to impaired cardiac function. In this study, we employed left anterior descending artery-ligated mice and oxygen‒glucose deprivation-induced cardiomyocytes to simulate MI in a clinical context. In the Ctrl group of MI mice, the anterior wall exhibited little contraction on the ultrasonic M-wave, the EF was approximately 40%, and considerable fibrosis and apoptosis were detected in the infarcted and peri-infarcted areas (Fig. 1). Similarly, in the OGD model, cardiomyocytes exhibited reduced cell viability and increased apoptosis. These results suggest the successful establishment of the animal and cell models.

To improve the clinical outcomes of MI patients, developing a safe and effective antiapoptotic approach is of critical significance [41]. The utilization of salidroside, could be a potential therapeutic strategy. Our previous study demonstrated that pretreatment with *R. sacra* mitigated exhaustive exercise-induced myocardial damage [18]. Additionally, salidroside can safeguard against cardiomyocyte apoptosis not only in cardiomyocytes exposed to oxygen‒glucose deprivation in vitro [15, 42] but also in mice with myocardial ischemia‒reperfusion or diabetes [43–45]. Our results are in line with these findings (Figs. 1 and 4). In this study, we administered salidroside at concentrations of 30 mg/100 mL and 60 mg/100 mL orally to mice. Salidroside is not genotoxic, with no evidence of adverse events reported in clinical contexts [46].

Moreover, in the current study, we affirmed that MIF was a target of salidroside. Studies have indicated that MIF is an endogenous regulator of MI that can effectively improve cell survival and heart function [19, 47]. Ruze et al. demonstrated that MIF could safeguard the heart from ischemic preconditioning-induced injury through the activation of the RISK pathway, AMPK, and membrane translocation of GLUT4 in mice [48]. Wu et al. reported that ischemia mimic treatment promoted the secretion of MIF via an autophagy-dependent pathway [49]. Similarly, our previous studies demonstrated that exercise could alleviate nonalcoholic fatty liver disease (NAFLD) by facilitating the AMPK/SIRT1 pathway and that *Rhodiola* treatment activated MIF and downstream lipophagy, as well as lipid metabolism, and subsequently reduced lipid accumulation in the liver in NAFLD [21, 50]. In this study, oxygen‒glucose deprivation led to cardiomyocyte death, which was characterized by the activation of the mitochondrial apoptosis pathway, and the use of si*Mif* had the same effect. Moreover, additional supplementation with MIF reversed these effects and increased p-AMPK expression (Fig. 5). These results imply that MIF plays a crucial role in oxygen‒glucose deprivation-induced apoptosis and that AMPK-mediated mitochondrial autophagy is a downstream regulatory pathway.

Traditional Chinese medicines are regarded as activators of cellular mitochondrial quality control, which involves fission, fusion, and autophagy [51]. Recent studies disclosed that salidroside serves as an autophagy activator. Zhu et al. incubated human umbilical vein endothelial cells (HUVECs) with oxidized low-density lipoprotein to establish a model of atherosclerosis and reported that salidroside enhanced autophagy in HUVECs and exerted protective effects [52]. Additionally, salidroside promotes gastric cancer cell apoptosis by upregulating the autophagy pathway [53]. Moreover, we formerly demonstrated that MIF is a key protein in the treatment of NAFLD with salidroside, and molecular docking, molecular dynamics, and SPR affirmed the binding activity between salidroside and MIF [21]. We further probed into the role of MIF in the salidroside-mediated alleviation of OGD-induced injury. We administered salidroside to the rMIF group or Mif gene-specific siRNA-transfected cells. Notably, salidroside in combination with exogenous rMIF supplementation decreased cell apoptosis and stimulated autophagy, while MIF silencing hindered most of the positive effects of salidroside (Fig. 6). These outcomes suggest that MIF is a target of salidroside.

Regarding *MIF* gene variations, there exist two distinct groups with high and low expression of MIF alleles [54]. This variation might influence the outcomes of cardiac surgeries and immunological diseases [55, 56]. The result concerning the correlation between *MIF* gene variations and this study could offer solid evidence for the personalized treatment of MIF and the utilization of salidroside as a potential therapy. Naturally, further research is requisite to examine this notion.

Despite the utilization of siRNAs, this study did not knockout the gene in mice to further clarify the effect of MIF. Moreover, exercise has been shown to activate MIF, and salidroside can imitate the effects of exercise. However, we did not compare a single intervention with salidroside combined with exercise. Further investigations may be conducted in the future.

## Conclusions

In conclusion, our findings reveal that salidroside can alleviate myocardial apoptosis and improve cardiac dysfunction in MI model mice. These effects might be related to the activation of the MIF pathway and the facilitation of downstream mitochondrial quality control. This study indicates that salidroside could be a potential drug for improve MI in clinical practice and offers new perspectives on its molecular mechanism.

## Supplementary Information

Additional file 1.

## Data Availability

The dataset supporting the conclusions of this article is available in the Figshare repository (https://doi.org/10.6084/m9.figshare.26507326).
