# Supplementary material for: Salidroside protects against myocardial infarction via activating MIF-mediated mitochondrial quality control
Source: Chin Med. 2025 Feb 28;20:27. doi: 10.1186/s13020-025-01076-3 (PMC11869418; doi:10.1186/s13020-025-01076-3)
Supplement: Supplementary file 1 — Additional file 1. [file 13020_2025_1076_MOESM1_ESM.pdf]

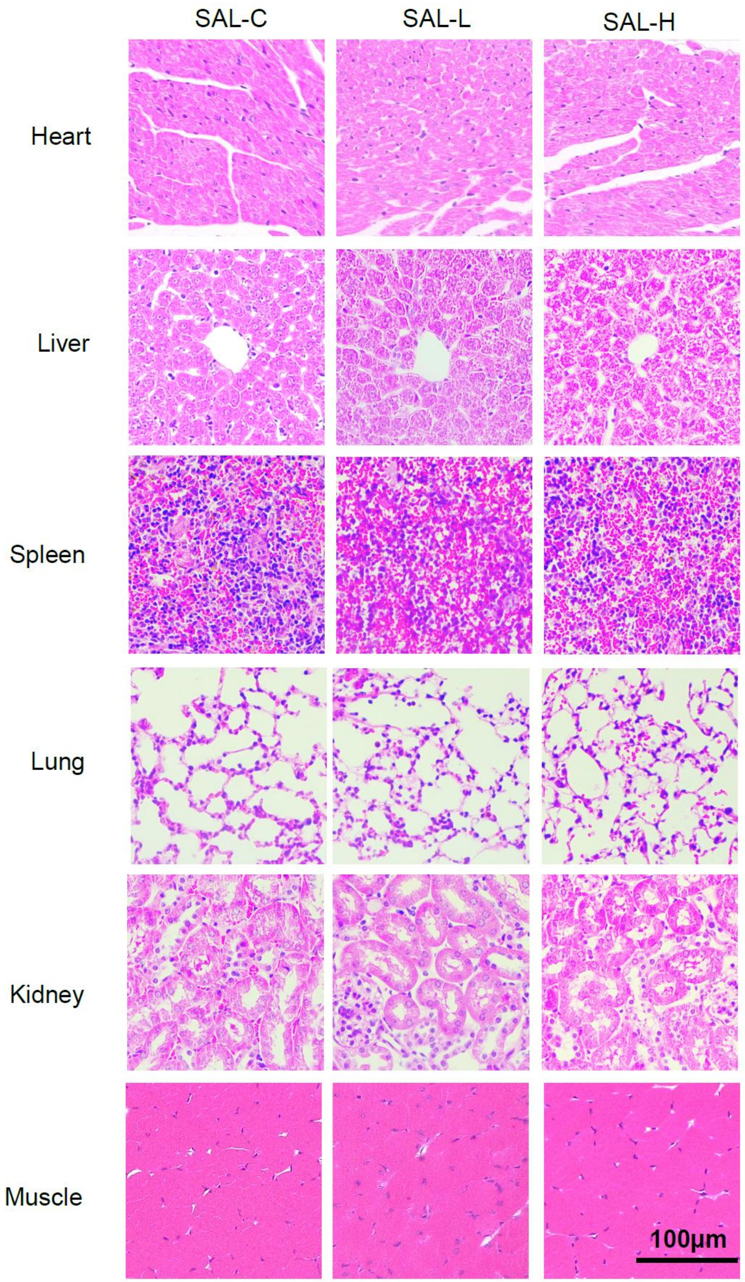

Fig. S1. salidroside toxic effect assessment.
Myocardial infarction mice were administered with vehicle control (Ctrl), or salidroside at a low dose (SAL-L, 100 mg/kg) or a high dose (SAL-H, 200 mg/kg) once per day. On day 28 post surgery, tissues were harvested to perform HE staining.

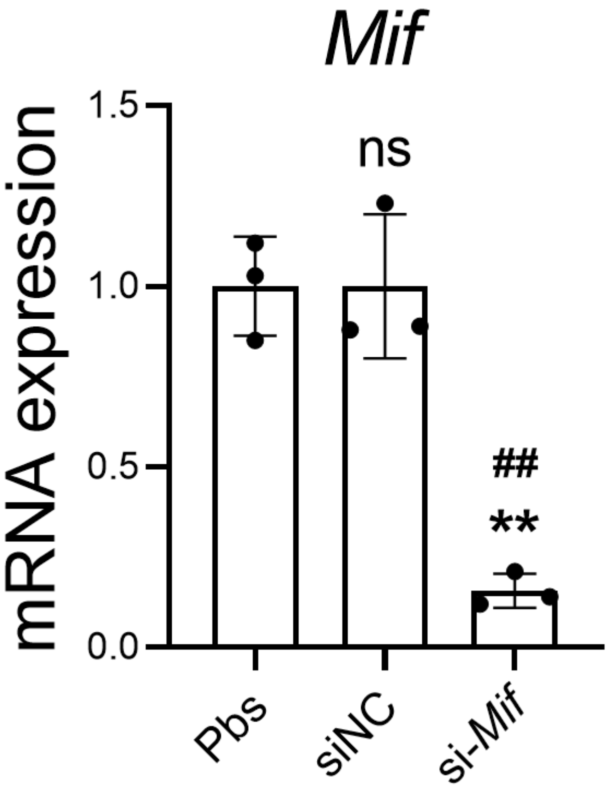

Fig. S2. si*MIF* transfection efficiency determine.
Cardiomyocytes were incubated with PBS or transfected with siNC or si*MIF*, then the *Mif* mRNA were assessed by qPCR.
